# Social cognition in multiple sclerosis and its subtypes: A protocol for systematic review and meta-analysis

**DOI:** 10.1097/MD.0000000000021750

**Published:** 2020-08-14

**Authors:** XiaoGuang Lin, XueLing Zhang, QinQin Liu, PanWen Zhao, JianGuo Zhong, PingLei Pan, GenDi Wang, ZhongQuan Yi

**Affiliations:** a Department of Neurology, Affiliated Suqian Hospital of Xuzhou Medical University, Suqian; b Department of Central Laboratory; c Department of Neurology, Affiliated Yancheng School of Clinical Medicine, Nanjing Medical University, Yancheng, Jiangsu Province, PR China.

**Keywords:** facial emotion recognition, meta-analysis, multiple sclerosis, protocol, social cognition, systematic review, theory of mind

## Abstract

**Background:**

Multiple sclerosis (MS) is an inflammatory and degenerative neurological disorder of the central nervous system. Cognitive impairment is frequent in MS patients, which not only includes deficits in abilities assessed by traditional neuropsychological batteries, but also often features impairments in social cognition (including theory of mind and facial emotion recognition). Recently, numerous studies have assessed social cognition performance in MS. However, there have been inconsistent findings. Besides, it is not clear how social cognitive abilities are affected in MS subtypes. The aim of this study is to conduct a meta-analysis to characterize social cognition performance in MS and its subtypes (clinically isolated syndrome, relapsing-remitting MS, progressive primary MS, and secondary progressive MS).

**Methods:**

Literature sources will be divided into 2 sections: electronic sources and manual sources. A systematic literature search will be performed for eligible studies published up to June 10, 2020 in 3 international databases (Embase, PubMed, and Web of Science). In addition, manual sources will be searched, such as the references of all included studies. Two researchers will independently conduct the work such as article retrieval, screening, quality evaluation, data collection. Meta-analysis will be conducted using Stata 15.0 software.

**Results:**

The results of this study will be published in a peer-reviewed journal.

**Conclusions:**

This meta-analysis will provide a high-quality synthesis from existing evidence for social cognition performance in MS and its subtypes.

**PROSPERO registration number:**

INPLASY202070028.

## 1 Introduction

Multiple sclerosis (MS) is an inflammatory and degenerative neurological disorder of the central nervous system.^[1]^ It is characterized by multifocal destruction of myelin sheaths and axonal loss.^[2,3]^ The course of MS is largely unpredictable, which overburdens patients together with motor disability and cognitive impairment.^[4]^ Based on the clinical course descriptions revised in 2013, MS phenotypes can be divided into relapsing and progressive diseases.^[5,6]^ Relapsing disease includes the clinically isolated syndrome (CIS) and relapsing-remitting MS (RRMS) subtypes. The CIS subtype refers to a first episode of MS-like neurologic symptoms that lasts at least 24 hours followed by complete or partial recovery. The RRMS subtype is the most frequent type of MS (80–85%), which is characterized by the onset of recurring clinical symptoms followed by total or partial recovery. Progressive disease includes progressive primary MS (PPMS) and the secondary progressive MS (SPMS) subtypes. The PPMS subtype accounts for 10% to 15% of MS, which is characterized by progressive accumulation of disability from the onset. The SPMS subtype follows the relapsing-remitting course, but the progression of disease is more stable, with or without superimposed relapses.

Cognitive impairment is frequent in MS patients. It not only includes deficits in abilities assessed by traditional neuropsychological batteries such as information processing speed, sustained attention, memory, and executive functioning,^[7,8]^ but also often features impairments in social cognition.^[9–12]^ Social cognition can be defined as “the mental operations that underlie social interactions, including perceiving, interpreting, and generating responses to the intentions, dispositions, and behaviors of others.”^[13,14]^ One of the aspects of social cognition is facial emotion recognition, which is the ability to identify and discriminate between the emotional states of others based on their facial expressions.^[15]^ Theory of mind (ToM), another aspect of social cognition, refers to the ability to attribute mental states to others, and to use the attributions to understand and predict behavior.^[16,17]^ As 2 core aspects of social cognition, facial emotion recognition and ToM collectively drive interpersonal skills, and may have important implications for social functioning.^[18,19]^

Recently, a number of studies have also investigated social cognition in MS.^[12,20–22]^ However, there have been inconsistent findings. For example, in aspect of facial emotion recognition, some studies found that compared to healthy controls (HC), patients with MS have difficulties only in recognition of the negative emotions of fear and anger.^[23,24]^ While Prochnow et al^[10]^ reported that relative to HC, MS patients were impaired in facial affect recognition on 4 of the 6 basic emotions, except happiness and disgust. Besides, Henry in 2009 et al^[25]^ found difference only in fear and surprise between MS patients and HC. In aspect of ToM, a number of studies have found that compared to HC, patients with MS have significant impaired in faux pas test (FPT, 1 ToM task),^[11,21,26]^ while Mike et al and Ouellet et al found no difference in FPT between MS patients and HC.^[20]^ These inconsistent findings might be related to the low statistical power as many of the available studies have small sample sizes. A meta-analysis can be helpful to increase statistical power, clarify conclusions of inconsistent findings in individual studies, and estimate the effect size for social cognitive deficits in MS.

To our knowledge, there are 2 recent meta-analyses that summarized social cognitive deficits between MS and HC.^[27,28]^ Bora et al^[27]^ and Cotter et al^[28]^ calculated the social cognition scores based on numerous very different ToM tasks and facial emotion recognition tasks. However, the quantitative results between MS and social cognition remain inconclusive. First, Bora et al^[27]^ found that compared to HC, patients with MS have medium impairment in Reading the Mind in the Eyes Test (RMET, 1 ToM task; d = 0.67), while Cotter et al^[28]^ found large impairment in RMET (d = 0.92). Second, Bora et al^[27]^ found relative to HC, MS patients significantly underperformed in all 6 basic emotions, but Cotter et al^[28]^ found MS patients were impaired only in anger, fear, and sad. Besides, previous meta-analyses calculated only 2 kinds of individual ToM tasks (FPT and RMET).^[27,28]^ Besides, it is important to investigate the diagnostic specificity of particular individual ToM tasks, as it is likely that some individual ToM tasks (such as false belief test [FBT] and strange stories test [SST]) may have a greater sensibility to detect mentalizing problems in MS. Moreover, previous meta-analyses separately analyzed social cognitive domains (including ToM and facial emotion recognition) only in RRMS.^[27]^ It is not clear how social cognitive abilities are affected in other MS subtypes (such as CIS, PPMS, and SPMS), as recently it has been reported that there are different patterns and severity levels of neurocognitive deficit between relapsing and progressive forms in MS.^[29,30]^

In view of these limitations in the previous meta-analyses, we conducted a systematic review and meta-analysis to systematically characterize social cognition performance in MS and its subtypes (CIS, RRMS, PPMS, and SPMS). In addition, we evaluated potential moderators of impairments observed in these individuals to help explain any variability between studies. Our meta-analysis will be helpful to promote understanding of social cognition in MS, which may be helpful for identification of targets for cognitive interventions and developing useful training intervention programs.

## 2 Methods

### 2.1 Study registration

We will conduct this systematic review and meta-analyses based on the guidelines of the Preferred Reporting Items for Systematic Reviews and Meta-Analyses Protocols (PRISMA-P) statement.^[31]^ This protocol has been registered in the International Platform of Registered Systematic Review and Meta-Analysis Protocols (INPLASY), and the INPLASY registration number is INPLASY202070028 (URL = https://inplasy.com/inplasy-2020-7-0028/).

### 2.2 Ethical approval

Ethical approval is not required because the data used in this paper are from published studies without the involvement of individual or animals experiments.

### 2.3 Criteria of selection for study

#### 2.3.1 Criteria for inclusion

Studies will be included if they met the following criteria: study design limited to case-control studies; the study should be published as a primary peer-reviewed research article in English; the study had to assess ToM or facial emotion recognition performance using standard measures; sufficient data to calculate effect sizes and standard errors of the ToM or facial emotion recognition were reported; a matched HC group had to be included.

#### 2.3.2 Criteria for exclusion

Studies will be excluded if they met the following criteria: the study lacked an HC group; the study with the patient samples was overlapped with another one with a larger sample size; the publication was not an original type, such as research protocols, letters, conference abstracts, reviews, and editorials; if the sample size of 1 study was under 10, the study will be excluded to ensure the reliability of the outcome.^[16]^

#### 2.3.3 Types of participants

Patients diagnosed with MS will be included in the study. Patients with other serious complications, a history of brain surgery, or other serious neurodegenerative diseases will be excluded from this study.

#### 2.3.4 Types of interventions

We will mainly study the performance of ToM and facial emotion recognition between MS patients and HC.

#### 2.3.5 Type of comparators

Healthy controls.

#### 2.3.6 Types of outcome measures

Primary outcomes will include the ToM tasks and facial emotion recognition tasks used. Besides, the data used for calculating the effect sizes and standard errors of the ToM/facial emotion recognition tasks will be included. Additional outcomes will include the questionnaire of clinical symptoms of MS.

### 2.4 Data sources

#### 2.4.1 Electronic searches

Three electronic databases (PubMed, Web of Science, and Embase) have to be searched from inception to June 10, 2020. There were no restrictions of the age of patients or phenotype of MS for inclusion. In addition, other resources will be searched manually, such as the references of all included studies.

#### 2.4.2 Search strategy

The search terms consist of 2 parts: MS and social cognition. The Medical Subject Headings and text words will be used in combination. The terms to be used in relation to MS include “multiple sclerosis” and “MS.” The terms to be used in relation to the social cognition include “social cognition,” “theory of mind,” “ToM,” “mentalizing,” “mentalising,” “facial emotion recognition,” and “emotion.” The search strategies are presented in Table 1.

**Table 1:**
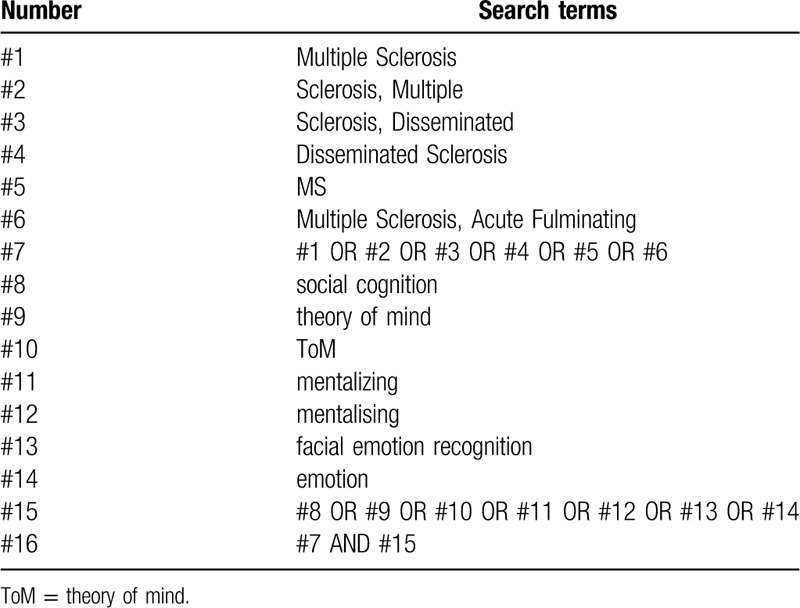
Search strategy for the PubMed database.

### 2.5 Data collection and analysis

#### 2.5.1 Selection of studies

**Figure 1:**
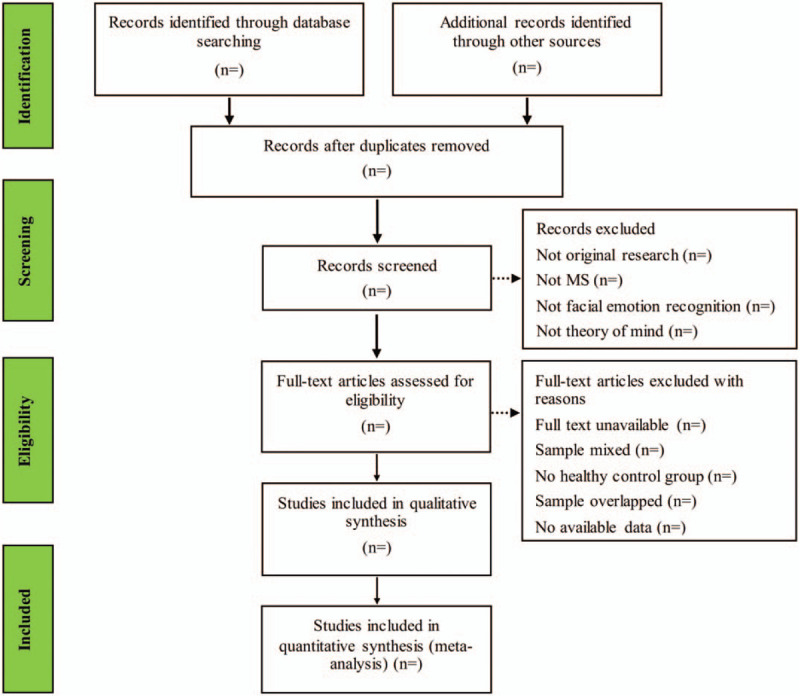
Flow diagram of studies search and selection.

The EndNote software, version X9 (United States) will be used to manage all literatures. Two investigators will independently review and screen the literatures in accordance with predetermined inclusion and exclusion criteria. Any disagreement will be discussed between the 2 reviewers, and further disagreements will be arbitrated by the third author. The process of selecting literature for the entire study is presented in the preferred reporting items for systematic review and meta-analysis (PRISMAP) flow diagram (Fig. 1).

#### 2.5.2 Assessment of quality in included studies

We will use the Newcastle-Ottawa Quality Assessment Scale to assess the quality of all included studies.^[32]^

#### 2.5.3 Data extraction and management

A unified data extraction form will be designed. Two investigators will independently extract data in the following domains: first author, publication year and title, inclusion/exclusion criteria, number of groups, number of participants, patients’ age, sex, education level, disease duration, MS phenotypes, healthy controls’ age, sex, education level, the facial emotion recognition tasks used, the data used for calculating the effect sizes and standard errors of the facial emotion recognition measures, the individual ToM tasks used, the data used for calculating the effect sizes, and standard errors of the ToM measures. Any disagreement will be discussed between the 2 investigators, and further disagreements will be arbitrated by the third author.

### 2.6 Data synthesis and statistical analysis

#### 2.6.1 Measures of treatment effect

Stata 15.0 software will be used for data analysis and quantitative data synthesis. The mean effect size (Hedges g) and 95% confidence intervals (CI) will be used to evaluate the performance of ToM and facial emotion recognition.^[33]^

#### 2.6.2 Dealing with missing data

For missing data, we will try to contact the first or corresponding authors of the included studies via email to acquire relevant information that is not available in the study. If the relevant data are not available after contacting the author, we will fully consider the associated risk of bias for missing data and use the available data to analysis.

#### 2.6.3 Data synthesis

As some studies did not provide a total mean score on ToM performance or included more than 1 individual ToM task, pooled effect size and standard error value were aggregated by computing the mean effect size.^[34]^ Similarly, the facial emotion recognition performance and social cognition performance were calculated.

#### 2.6.4 Assessment of heterogeneity

We will assess the heterogeneity by I2 statistics. ^[35]^ base on a standard linear hypothesis with I^2^ < 50 indicating low heterogeneity.^[35]^ If I^2^ value is less than 50%, we will apply fixed-effects model to homogeneous data; otherwise, the random-effects model will be applied.

#### 2.6.5 Assessment of publication bias

We will use funnel plots to detect publication bias. If the analysis includes ≥10 studies in meta-analysis, a test for funnel plot asymmetry using Egger method will be conducted.^[36]^

#### 2.6.6 Sensitivity analysis

We will conduct a sensitivity analysis to assess the reliability and robustness of the aggregation results via eliminating trials with high bias risk. If reporting bias was found, we will apply the trim-and-fill method to provide effect sizes adjusted for publication bias.^[37]^

#### 2.6.7 Subgroup analysis

Subgroup analysis will be performed in individual ToM tasks (such as FPT, RMET, FBT, SST), 6 basic motions (such as anger, disgust, fear, happy, sad, and surprise), and MS subtypes (such as CIS, RRMS, PPMS, and SPMS).

#### 2.6.8 Meta-regression analysis

If data are available, we will conduct meta-regression analyses to investigate social cognition performance with reference to various factors including the age, gender, education level, and disease duration, with a random-effects model using the restricted-information maximum likelihood method with the significance level set at *P* < .05.

## 3 Discussion

Social cognitive deficits are an important aspect of cognitive impairment in MS, which may have potential prognostic significance for social functioning and quality of life. Previous studies found social cognitive deficits may be comparable in magnitude to or even exceed other neurocognitive impairments and should also be incorporated into routine neurologic assessments.^[28]^ This meta-analysis will be helpful to promote understanding of social cognition in MS. Our results may emphasize the need to increase awareness among treating physicians of social cognitive dysfunction. Social cognitive training has been shown to be effective in other disorders^[38]^ and it is hoped that our result can be helpful for informing the development of similar interventions for those with MS.

## Author contributions

**Conceptualization:** XiaoGuang Lin, XueLing Zhang.

**Data curation:** QinQin Liu, PanWen Zhao.

**Investigation:** XiaoGuang Lin, PanWen Zhao.

**Methodology:** PingLei Pan, JianGuo Zhong.

**Supervision:** PingLei Pan, JianGuo Zhong.

**Validation:** GenDi Wang.

**Writing – original draft:** XiaoGuang Lin, GenDi Wang.

**Writing – review & editing:** ZhongQuan Yi, GenDi Wang.
